# Integrating hemodynamic analysis with traditional imaging in intracranial atherosclerotic stenosis: current status and future perspectives

**DOI:** 10.3389/fneur.2025.1589162

**Published:** 2025-07-18

**Authors:** Xulong Yin, Rui Yang, Zhen Li, Hui Wang, Qi Fang

**Affiliations:** ^1^Department of Neurology, The First Affiliated Hospital of Soochow University, Suzhou, Jiangsu, China; ^2^Institute of Stroke Research, Soochow University, Suzhou, China

**Keywords:** intracranial atherosclerotic stenosis, computational fluid dynamics, diagnosis, hemodynamics, neuroimage

## Abstract

Intracranial atherosclerotic stenosis (ICAS) is a significant cause of ischemic stroke. Traditional imaging methods have their own advantages and disadvantages in the diagnosis of ICAS. Hemodynamic analysis technology, as a new technique and method based on conventional imaging examinations, when combined with traditional imaging, can more comprehensively assess the hemodynamic status of ICAS patients, providing a new direction for the diagnosis and research of ICAS. This review provides an overview of the traditional imaging techniques currently used to diagnose ICAS, including transcranial Doppler ultrasound (TCD), computed tomography angiography (CTA), magnetic resonance angiography (MRA), high-resolution magnetic resonance imaging, and digital subtraction angiography (DSA). The unique characteristics of each method are elaborated. In addition, examples of hemodynamic analysis applications based on these traditional imaging techniques are discussed. This article summarizes and reviews the prospects and limitations of ICAS hemodynamic analysis and proposes potential directions for future research. As a recently developed clinical evaluation method for ICAS, hemodynamic analysis techniques have demonstrated significant potential in various aspects of ICAS, including clinical diagnosis, etiological exploration, treatment selection, and recurrence prediction. It is hoped that the method of hemodynamic analysis will ultimately be incorporated into the treatment guidelines for ICAS patients, paving new ways for the diagnosis and treatment of ICAS.

## 1 Introduction

Intracranial atherosclerotic stenosis (ICAS) is one of the most common causes of ischemic stroke or transient ischemic attack (TIA) worldwide (1). Annual mortality rates were reported to be 12.4% per year for intracranial internal carotid artery stenosis, 6.8% for middle cerebral artery (MCA) stenosis, and 11.6% for vertebrobasilar stenosis (2). Moreover, patients with symptomatic intracranial atherosclerotic stenosis (sICAS) are also at a higher risk of recurrent ischemic stroke, up to 25–30% in 2 years after an index stroke (3–5). These statistics underscore the pressing need for improved understanding and management strategies that may reduce both stroke incidence and associated mortality (6).

Diagnosis of ICAS is divided into invasive and non-invasive methods. Several non-invasive screening tests such as transcranial doppler (TCD), computed tomography angiography (CTA), magnetic resonance angiography (MRA), and high-resolution magnetic resonance imaging (HR-MRI), which can evaluate the condition of intracranial blood vessels more safely and economically (7–9). As for invasive test, digital subtraction angiography (DSA) remains the gold standard for ICAS diagnosis and provides fundamental information about blood flow dynamics, the extent of intracranial vascular stenosis, and collateral circulation in patients (6). Currently, radiographic risk assessment is primarily based on the severity of anatomic stenosis, but the percentage of luminal stenosis represents only one aspect of ICAS. Many elements may be related to the prognosis of symptomatic ICAS, such as plaque features, downstream perfusion status, and collateral branches (8, 10–12). Misjudging the risk of stroke recurrence solely based on the maximum degree of lumen stenosis, without considering hemodynamic characteristics, may lead to serious consequences (12).

Hemodynamic analysis in ICAS development is inspired by cardiovascular diseases. In 1993, Pijls introduced a new parameter for estimating coronary blood flow through pressure measurement: Fractional Flow Reserve (FFR) (13), which is a ratio of the maximal flow in a stenotic artery compared to the maximal flow of abnormal artery (14). Through gradual development, FFR-guided percutaneous coronary intervention (PCI) has gradually become the standard recommended by guidelines for assessing the hemodynamic significance of coronary artery stenosis (15). Hemodynamic analysis has gradually been introduced into the study of intracranial atherosclerosis, and research on FFR in the field of neurointervention has made it possible to use invasive FFR wire measurements in ICAS (16).

In recent years, the non-invasive blood flow assessment method of ICAS has distinguished itself when compared with invasive measurements (via pressure wire through stenosis). As a representative method for hemodynamic analysis, Computational Fluid Dynamics (CFD) has gained increasing applications in biomedical research due to its high-performance hardware and software. CFD technology has gradually achieved results comparable to invasive measurements and is expected to assist in evaluating the functional changes of cerebral vascular stenosis (17). Multiple studies have demonstrated the feasibility of hemodynamic analysis in ICAS research, and many related hemodynamic indicators such as pressure ratio (PR), wall shear stress (WSS), fractional pressure ratios (FPR), quantitative flow ratio (QFR), translational pressure difference are used as references (16–21). These hemodynamic indicators enrich the system of hemodynamic analysis for ICAS, reigniting the enthusiasm for research in the etiology, diagnosis, and treatment of ICAS diseases.

This review aims to provide a comprehensive overview of hemodynamic analysis in the context of traditional imaging modalities for ICAS. We examine how these imaging-based hemodynamic parameters can aid in disease characterization, treatment planning, and prognostication. By summarizing recent developments and highlighting gaps that warrant further investigation, we hope to offer insight into potential future directions for ICAS research and clinical management.

## 2 Hemodynamic analysis and computational fluid dynamics

Hemodynamic analysis refers to the quantitative analysis of various kinetic parameters during blood flow. The application of hemodynamic analysis in the cervical and cerebrovascular vessels has gone through a long process. The relationship of cervico-cerebral arterial stenosis and cerebral blood flow (CBF) was first postulated by Spencer and Reid (22). They proposed that carotid artery stenosis of 70% will not lead to a substantial decrease in cerebral blood flow, while when the stenosis reaches 80%, cerebral blood flow will decrease sharply. However, other factors such as the effects of turbulence, collateral circulation, altered distal flow resistance, are not considered in such hypotheses, each of which may complicate the relationship between vascular stenosis severity and CBF *in vivo* (23). This has stimulated the further application and exploration of hemodynamic analysis in cerebral hemodynamics.

As the predominant and systematic approach to hemodynamic research, CFD modeling bridges experiments with theory (24), simulating flow patterns by solving fluid dynamics equations, and serves as a valuable tool for investigating hemodynamics (25). The establishment of the fluid mechanics model in ICAS can be roughly divided into the following six steps (26). (1) Image Acquisition: obtain high-quality images often via modalities such as CTA, MRA, or DSA to visualize the target vessels. (2) Model Construction: reconstruct a three-dimensional (3D) geometric model of the vessel lumen from the acquired images. (3) Data Discretization: convert continuous spatiotemporal blood flow data into discrete elements (meshing) and time steps, facilitating numerical computation. (4) Boundary Condition Setting: define inlet and outlet conditions (e.g., flow velocity, pressure), vessel wall properties, and fluid characteristics (blood density, viscosity). These parameters must approximate physiological conditions within the cerebrovascular system. (5) Computational Solving: use numerical methods to solve the Navier–Stokes equations under the defined boundary conditions to obtain parameters such as velocity fields, pressure gradients, and wall shear stress. (6) Validation: compare the simulation results with *in vitro* experiments or *in vivo* measurements to ensure model accuracy and reliability. In recent years, CFD modeling based on conventional neurovascular imaging has been applied to simulate in vivo cerebral blood flow and quantify cerebral hemodynamic metrics in the presence of ICAS, which cannot be achieved with conventional neurovascular imaging alone (27, 28).

## 3 ICAS traditional imaging development and combination of hemodynamic analysis

### 3.1 Transcranial Doppler ultrasound

In 2002, Moehring et al. introduced the multi-gate power-motion Doppler (PMD), an ultrasound detection mode that replaced the single-gate spectral transcranial doppler (TCD) introduced by Aaslid et al. (29, 30). By simplifying the scanning procedure, this advancement contributed to the broad adoption of TCD in various clinical settings (31). TCD is recognized for its safety, affordability, and ease of use (32). In addition, it allows for the detection of microembolic signals and facilitates vasomotor reactivity quantification, both of which help predict ischemic stroke recurrence in patients with ICAS and reflect the brain’s capacity for autoregulation (33). Nonetheless, diagnostic performance may be hindered by factors such as an inadequate temporal bone window, suboptimal insonation angles, or low flow velocity/volume (34). Despite continuous improvements in TCD technology for ICAS evaluation, its accuracy and predictive value remain dependent on operator proficiency and patient-specific characteristics.

TCD is also central to the hemodynamic assessment of ICAS, given its ability to measure and monitor cerebral hemodynamics in real time (35). Wijnhoud et al. demonstrated that mean flow velocity (MFV) and the ratio of pulsatility index (PI) to MFV in the MCA are independent prognostic factors for stroke recurrence within 2 years in patients with minor ischemic stroke or TIA (36). Additionally, TCD can be employed to measure cerebrovascular reactivity (CVR) using breath-holding maneuvers. In the Mechanism of Early Recurrence in Intracranial Atherosclerotic Disease (MyRIAD) study, nearly 70% of patients with symptomatic ICAS exhibited low CVR (defined as a TCD breath-holding index <0.69) (37).

Building on TCD technology, transcranial color Doppler ultrasound (TCCS) integrates blood flow velocity measurement with parenchymal structure imaging, thereby enhancing the scope of hemodynamic analysis in ICAS (38). Recent TCCS-based investigations report sensitivities of 72.9–88.9%, specificities of 82.9–94.8%, positive predictive values of 51.1–79.4%, and negative predictive values of 77.3–99.3% for detecting vascular stenosis or occlusion (39, 40). By comparing transcranial contrast-enhanced color-coded sonography (CE-TCCS) with digital subtraction angiography (DSA), Liu et al. demonstrated that CE-TCCS can improve the visualization of intracranial vessels and accurately diagnose MCA stenosis (41). Moreover, Xu et al. showed that the PI obtained via TCD may reflect cerebrovascular resistance and changes in cerebral blood flow, corroborating its correlation with the flow pattern ratio (FPR) measured by pressure wire (42). Consequently, TCD in tandem with hemodynamic analysis offers distinct advantages for the screening and diagnosis of ICAS.

In addition to its role in static hemodynamic assessment, TCD is extensively employed for the evaluation of cerebral blood flow autoregulation (CBF-AR), a critical mechanism that maintains stable cerebral perfusion even in the presence of fluctuations in systemic blood pressure. A commonly utilized protocol for CBF-AR assessment involves the simultaneous acquisition of beat-to-beat arterial blood pressure, achieved through continuous finger blood pressure monitoring to capture real-time blood pressure variations. This is combined with TCD measurements of cerebral blood flow velocity (CBFV) in the middle cerebral artery, and the recording of end-tidal carbon dioxide concentrations using an infrared capnograph or mass spectrometer. This comprehensive approach allows for a dynamic and non-invasive evaluation of cerebrovascular reactivity and autoregulation in patients with ICAS (43–45). Such integrated methods are of significant value for risk stratification and treatment decision-making in ICAS patients.

### 3.2 Computed tomography angiography

Early applications of computed tomography angiography (CTA) demonstrated its utility in managing intracranial aneurysms (46, 47) and assessing carotid artery disease (48) and renal artery stenosis (49). As spatial resolution improved, CTA emerged as an effective tool for detecting intracranial arterial stenosis and occlusion (40, 50–53). When benchmarked against digital subtraction angiography (DSA), CTA achieves 100% sensitivity and specificity for identifying complete occlusion of major arteries. For stenosis of 50% or greater, CTA yields a sensitivity of 97.1%, specificity of 99.5%, and negative predictive value of 99.8%. Notably, in cases of reduced flow or turbulence distal to stenosis or occlusion, CTA tends to show superior vascular patency compared to DSA (53). Furthermore, CTA has exhibited good agreement with histopathology and intravascular ultrasound in distinguishing calcified plaques, intermediate plaques, and soft plaques (54). When combined with computed tomography perfusion (CTP), CTA can also assess ischemic lesions and localized hypoperfusion (55). Nonetheless, several limitations persist, including radiation exposure, the risk of contrast-induced nephropathy and allergic reactions, partial loss of laminar flow data, and the potential for the intracranial internal carotid artery to appear artificially narrowed or invisible near the cavernous sinus due to susceptibility artifacts (6).

CTA-based hemodynamic analysis is increasingly applied to ICAS, offering novel perspectives on stroke recurrence. In one study, Leng X found that hemodynamic parameters obtained from CFD models reconstructed using routine CTA data could predict stroke recurrence in symptomatic ICAS patients with 70–99% luminal stenosis (56). In a 2019 multicenter, large-cohort investigation correlating hemodynamic parameters with stroke recurrence risk in moderate-to-severe stenosis, patients with large cross-lesion pressure gradients and markedly elevated WSS on plaques were at higher risk of recurrent ischemic stroke, suggesting a potentially more effective predictor than traditional measures (57). Feng X subsequently employed CTA-based CFD to reveal that low systolic blood pressure (SBP) might elevate the risk of stroke recurrence in sICAS patients with high translesional pressure gradients (58), indicating the need for carefully tailored blood pressure management. In 2023, Feng X further showed that low perfusion is commonly linked to artery-to-artery embolism (AAE), while high WSS in intracranial atherosclerotic disease (ICAD) may increase AAE risk—thereby identifying a potential target for secondary stroke prevention (59). Tian X integrated conventional vascular risk factors with hemodynamic metrics in CFD models to develop the “D2H2A” nomogram, enabling risk stratification for recurrent stroke in sICAS patients (60).

The status of leptomeningeal collaterals (LMCs) circulation in ischemic stroke patients determines their prognosis. Leng X suggests a correlation between translational pressure gradients and the maturity of LMC in intracranial atherosclerotic disease. Further research is needed for more refined and dynamic monitoring of cerebral hemodynamics and LMCs evolution to validate current findings (61). In a related study, Leng X further demonstrated that pressure ratio (PR) and LMCs status are interlinked, with leptomeningeal collaterals and basal cistern collaterals jointly supporting distal perfusion in the setting of severe arterial stenosis (62). Overall, the integration of CTA and hemodynamic analysis is steadily expanding in both diagnosing and treating ICAS, underscoring its growing clinical and investigative importance.

### 3.3 Magnetic resonance angiography

Time-of-flight magnetic resonance angiography (TOF-MRA) was initially employed primarily for intracranial and cervical vascular imaging in its early stages (63, 64). Currently, MRA not only provides anatomical details but also captures blood flow information, making it increasingly favored for intracranial artery evaluations (6). With the advent of higher-field MRI scanners and advances in post-processing techniques, MRA’s diagnostic performance has been continuously enhanced (65–68). When benchmarked against digital subtraction angiography (DSA), TOF-MRA demonstrates a sensitivity of 78–85%, a specificity of 95%, a positive predictive value (PPV) of 75–79%, and a negative predictive value (NPV) of 95–97% for detecting high-grade stenosis (50–99%) (66). Nevertheless, TOF-MRA is highly susceptible to flow-related artifacts, which can cause a total loss of signal even in incompletely occluded vessels (69). It is also less suitable for assessing in-stent intracranial stenosis due to artifacts from stents or coils (6). Additional limitations include lower spatial resolution, which can impede accurate delineation of severe stenoses in small vessels or lead to overestimation of stenosis severity because of flow velocity or turbulence (65). Despite these drawbacks, MRA has the advantage of avoiding radiation exposure, and its diffusion-weighted imaging (DWI) component offers high sensitivity in the acute phase of stroke. Consequently, MRA remains a valuable screening modality for detecting intracranial atherosclerotic stenosis and occlusion (70).

Combining MRA with hemodynamic analysis has become increasingly common in the study of ICAS. Chen et al. reported correlations among ICAS stenosis severity, wall shear stress ratio (WSSR), and pressure ratio (PR) derived from MRA (28), thus laying a foundation for subsequent MRA-based hemodynamic research. In patients with ischemic stroke caused by atherosclerotic MCA stenosis, Wu et al. found that parameters such as WSSR are associated with functional outcomes (71). Using a combination of MR-based CFD and DSA, Roach et al. demonstrated that collateral vessels in patients with intracranial atherosclerosis may exhibit fundamentally different vascular reactivity compared with those in healthy vessels (72). Additionally, Kaczmarz et al. employed perfusion- and oxygenation-sensitive single-watershed area (iWSA) imaging to assess hemodynamic compromise in ICAS, revealing significant impairments in cerebral blood flow (CBF), cerebrovascular reactivity (CVR), relative cerebral blood volume (rCBV), and oxygen extraction fraction (OEF/CTH) (73).

**Figure 1:**
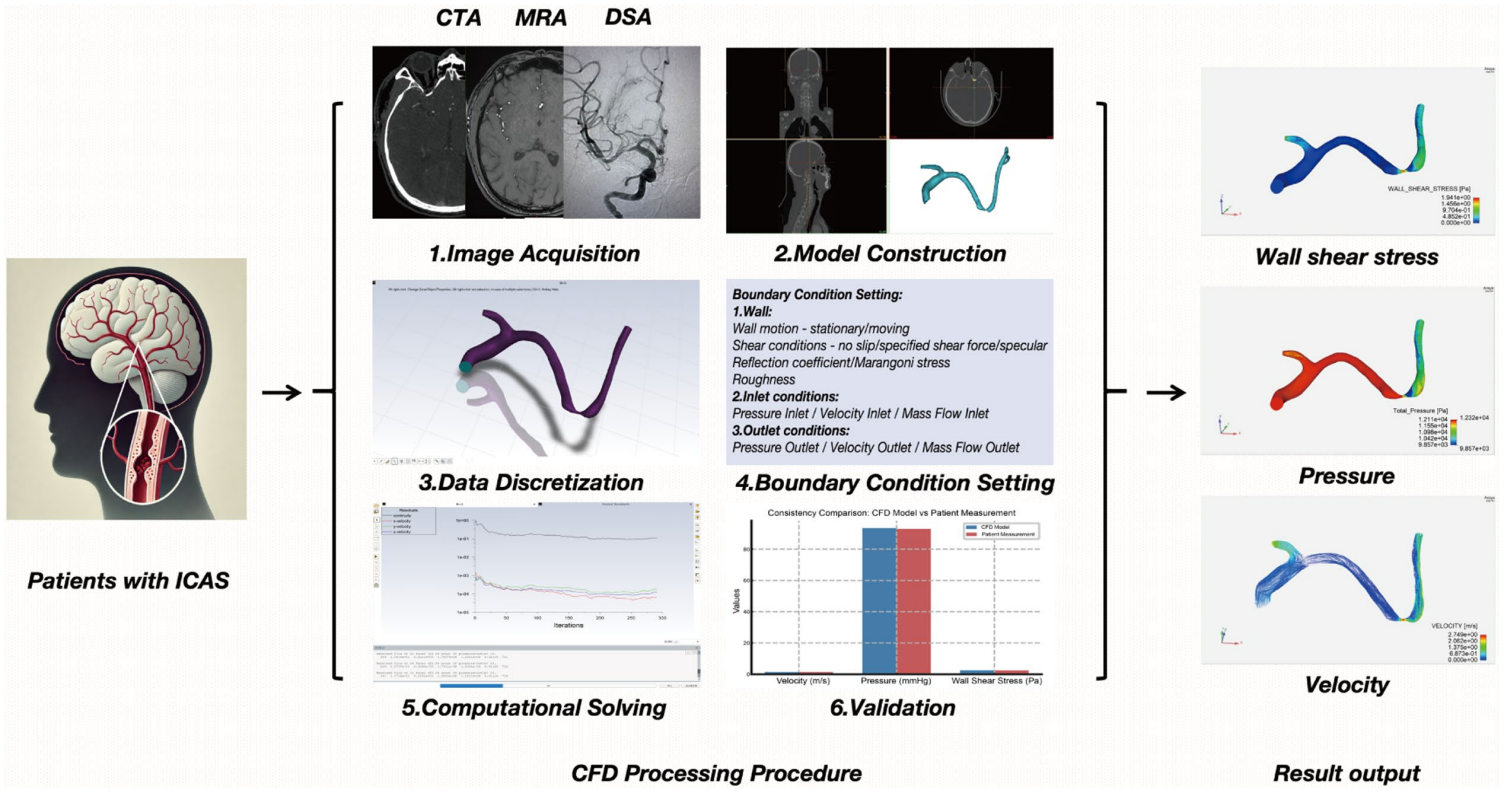
Flowchart illustrating the computational fluid dynamics analysis process.

**Figure 2:**
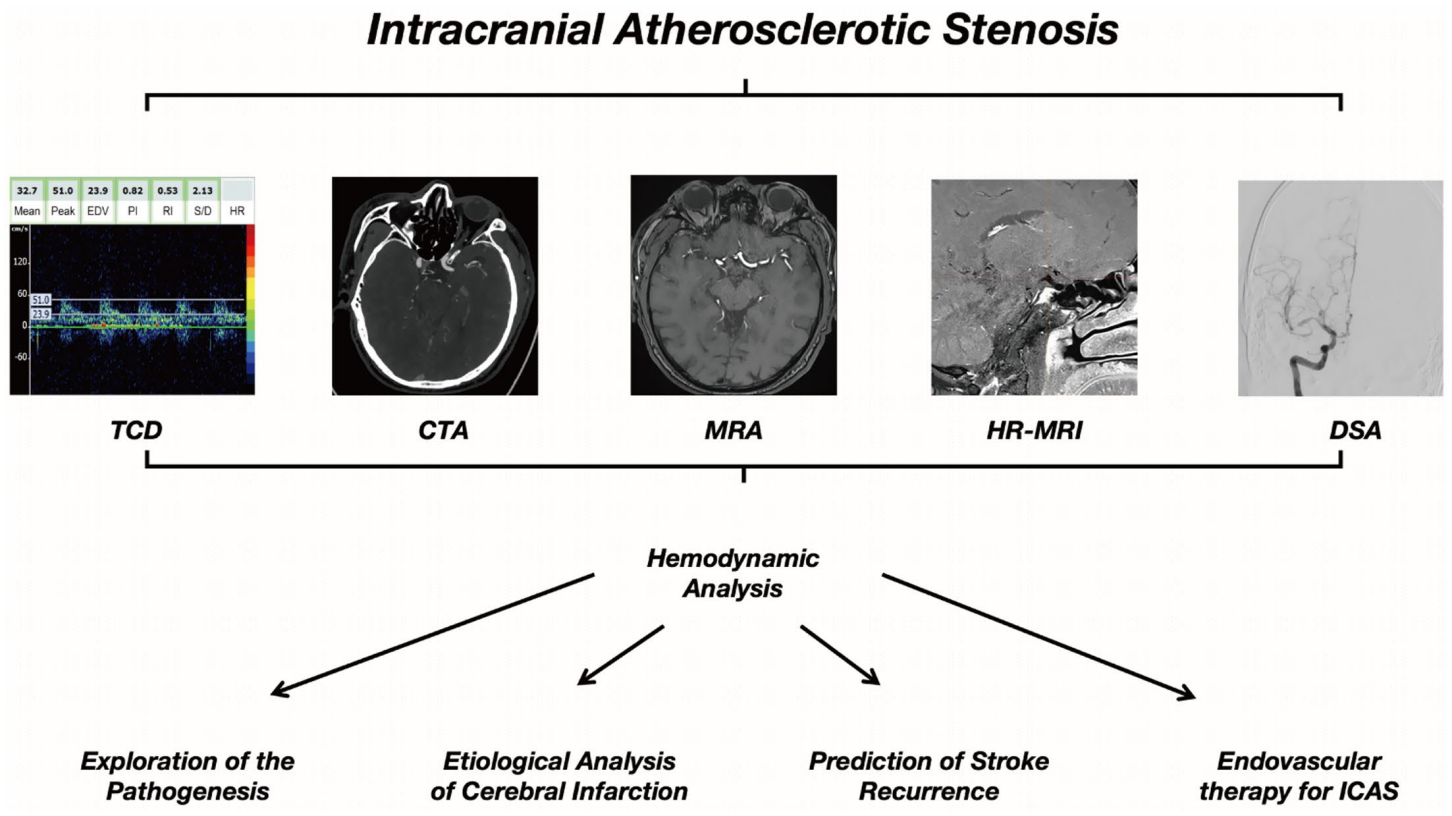
Schematic diagram of hemodynamic analysis in traditional imaging.

As research on MRA-based hemodynamic analysis progresses, changes in MRA signal intensity (SI) have been implicated as a potential reflection of the severity of hemodynamic impairment in ICAS (74), which has laid the foundation for subsequent studies. An index called the signal intensity ratio (SIR) has been developed to quantify the hemodynamic significance of ICAS in TOF-MRA, which has been shown to have high intra- and inter-observer consistency (75). In a group of patients with unilateral MCA stenosis, the SIR values were significantly lower in those with stage II and III cerebral hypoperfusion compared to those with normal perfusion (76). Subsequent studies have also found that the average SIR of symptomatic MCA stenosis is lower than that of asymptomatic MCA stenosis (77). In patients with intracranial ICA or MCA stenosis and continuous CBF in CTP, a lower SIR is significantly associated with prolonged or delayed perfusion, manifested as higher ipsilateral mean transit time (MTT) and ipsilateral/contralateral MTT ratio. All these studies support the value of SIR in evaluating the hemodynamic significance of ICAS (78). The WASID trial focused on predicting stroke risk, an SIR <0.9 was independently associated with an increased risk of recurrent stroke in the same field, with patients with 50 to 99% stenosis of a sICAS (adjusted HR [aHR] 10.9, 95% CI 2.0–58.9; *p* < 0.001) (79). Other studies have also used a simple method of evaluating changes in ICAS spanning the SI using TOF-MRA, by grading the visibility of ICAS in TOF-MRA, decreased visibility of distal MCA branches in patients with symptomatic unilateral MCA main stem stenosis (70 to 99%) is associated with the presence of internal borderzone infarction and increased risk of stroke recurrence (80). The application of the ICAS hemodynamic analysis method and process on MRA and CTA exhibits certain similarities and homologies (Figures 1, 2).

### 3.4 High-resolution magnetic resonance imaging

High-Resolution magnetic resonance imaging (HR-MRI) diagnosis of intracranial atherosclerotic plaques has a sensitivity of 85% and a specificity of 92% for burden and vulnerability (81). It not only indicates the degree of stenosis, but also shows intraplaque factors including plaque morphology, plaque composition, and inflammation, which are predictors of vulnerable plaques. Compared with stable plaques, vulnerable plaques have thinner fibrous caps, larger lipid nuclei, and a myriad of inflammatory cells, which are associated with forward remodeling, greater plaque thickness or higher plaque thickness to significant luminal tube ratio, intraplaque hemorrhage, and enhanced HR-MRI contrast (82). Vessel wall magnetic resonance imaging (VWMRI), as a special category of HR-MRI, is one of the best non-invasive methods for displaying the characteristics of intracranial atherosclerotic vessels. The main clinical applications of this technique include the exploration of the pathogenesis of intracranial atherosclerotic lesions, follow-up monitoring, and treatment prognosis judgment (83). Unlike internal carotid artery VWMRI, intracranial artery VWMRI is still unavailable for obtaining in-vivo plaque pathology specimens for imaging-pathology control. VWMRI requires longer imaging times and has higher economic costs, limiting its application under specific conditions (84).

Hemodynamic analysis can also be used in combination with HR-MRI in ICAS studies. Zhang D attempted to use HR-MRI for CFD modeling, further improving the accuracy of the model, studying the blood flow dynamics changes in ICAS-induced vascular remodeling, and dividing patients into positive remodeling group and negative remodeling group, calculating relevant hemodynamic parameters. The study showed that WSSR, stenosed wall WSS, and plaque area were related to vascular remodeling. The degree of remodeling and plaque area were positively correlated with WSSR, reflecting that hemodynamic changes can cause intracranial vascular remodeling and changes in plaque characteristics, and patients with high hemodynamic parameters such as WSSR have a higher incidence of stroke (85). Li Z combined HR-MRI with hemodynamics analysis to study the potential relationship between the geometric morphology of the basilar artery (BA) and the distribution of atherosclerotic plaques. It was found that the inner arc of the tortuous BA with atherosclerotic disease was more likely to develop plaques, and the increased tortuosity of the BA was associated with the likelihood of plaque formation (86). Woo, H.G. compared the differences in plaque characteristics and hemodynamic parameters in the patients with MCA atherosclerotic to understand the mechanism of stroke. It was found that artery-to-artery embolic stroke was related to plaque enhancement and maximum WSS at the upstream point of the plaque, as well as the increased variability in maximum WSS (87). The methods and processes for ICAS hemodynamic analysis on MRA parallel those employed with CTA, underscoring their complementary roles in both research and clinical practice.

### 3.5 Digital subtraction angiography

Digital subtraction angiography (65) is considered the gold standard for quantifying intracranial atherosclerotic stenosis and evaluating collateral blood flow (5, 88) because of the inherent high spatial resolution leading to high imaging quality. However, disadvantages of DSA include high costs, limited availability, and a small risk (<1%) of serious periprocedural complications (89). The application of DSA in hemodynamic analysis of ICAS draws on the experience from coronary arteries. Han Y and Miao Z perform pressure measurements on the stenosis of patients with ICAS by means of a pressure guidewire (18, 90). In both studies, no relevant complications were encountered during the exploration of the pressure wire, and good pressure signals were obtained, demonstrating the safety and feasibility of measuring PR using a pressure wire. However, using pressure-guided wires is expensive, and due to the fragility of intracranial arteries, untrained surgeons may increase perioperative complications. Wang M proved that the PR calculated using CFD and pressure wire has good correlation and consistency (91). If combined with the CTP brain tissue ischemia threshold standard (Tmax >6 s), a PR cut-off value of 0.67 can be obtained, which is similar to the measurement results of pressure guidewires such as Han Y and Miao Z and can be used as a new non-invasive calculation method to quickly calculate PR from DSA without measuring pressure filaments.

DSA-based CFD also has a new exploration for the endovascular treatment of ICAS. For patients after ICAS stenting, Zhang K proved that suboptimal reperfusion of severe MCA stenosis can significantly improve the hemodynamic status of cerebral perfusion at the stenotic site (92). Song X found that stent implantation in ICAS patients altered the original vascular anatomy and affected local hemodynamics. Both the PR and the WSSR correlated linearly with the degree of vascular distortion, and ICAS lesions with lower PR in the lesion after stent placement showed a higher rate of restenosis (93). Huang K used quantitative digital subtraction angiography (q-DSA) to evaluate the predictive value of hemodynamic characteristics of blood flow after percutaneous transluminal angioplasty and stent implantation (PTAS) in patients with intracranial vertebral basilar artery atherosclerotic stenosis for recurrent stroke (94). Wu’s research integrates artificial intelligence algorithms to automate the delineation of vascular contours and calculate μQFR for the functional assessment of lesions in ICAS patients (95). The CFD model based on DSA avoids the influence of pressure guidewire on the lesion site and adds hemodynamic parameters as an aid based on the traditional DSA gold standard, which has a broader application prospect for the diagnosis and treatment of ICAS. The differences between ICAS and traditional imaging techniques are illustrated in Table 1.

**Table 1:** Characteristics of different imaging diagnoses for intracranial atherosclerotic stenosis.

Imaging category Characteristics	Transcranial Doppler ultrasound	Computed tomography angiography	Magnetic resonance angiography	High-resolution magnetic resonance imaging	Digital subtraction angiography
Imaging features	Real-time detection of blood flow velocity and blood flow direction, detection of microembolic signals and blood theft, detection of vascular reactivity.	Detection of luminal narrowing through visualization of arterial cavities, providing information on vascular calcification, plaque morphology, and collateral vessels.	Depicts and visualizes the arterial lumen based on the so-called inflow effect of unsaturated spins.	Demonstrate the intracranial arterial wall and degree of stenosis, identify plaque composition within the vascular wall, plaque conformity, vulnerability, and remodeling patterns.	Observation of hemodynamic status, measurement of intracranial vascular stenosis, and assessment of collateral circulation richness.
Invasive or non-invasive	Non-invasive	Non-invasive	Non-invasive	Non-invasive	Invasive
Accuracy	Sensitivity of 72.9–88.9%, specificity of 82.9–94.8%, PPV of 51.1–79.4%, and NPV of 77.3–99.3%.	Sensitivity and specificity of 100% in detecting complete occlusion of major arteries. In detecting narrowing greater than or equal to 50%, CTA demonstrates a sensitivity of 97.1%, specificity of 99.5%, and negative predictive value of 99.8%.	Sensitivity of 78 to 85%, specificity of 95%, PPV of 75 to 79% and NPV of 95 to 97% in detecting high-grade stenosis (50 to 99%).	Sensitivity of 85% and a specificity of 92% for burden and vulnerability.	Gold Standard.
Advantages	Safety, Economy, Convenience.	Accurate, efficient, and minimally invasive; boasting higher acquisition speed than MRA and less susceptibility to motion artifacts; can be combined with CTP; beneficial for distinguishing the nature of plaques.	No radiation exposure required; diffusion-weighted imaging (DWI) sequences offer higher resolution for acute cerebral infarction.	Differentiation of causes of vascular stenosis, clarification of stroke mechanisms, and guidance for treatment strategies.	High spatial resolution and high-quality images, regarded as the gold standard.
Disadvantages	Dependent on the technical proficiency of the ultra sonographer and the varying anatomical conditions of the patient.	Risks associated with radiation exposure, the use of iodinated contrast agents, and the lack of laminar blood flow dynamics information.	Overestimation of stenosis severity due to blood flow-related artifacts; unfavorable for post-stent assessment.	Invasive procedures, radiation exposure, cost, and certain risks such as kidney damage from contrast agents, allergies, and ischemic stroke.	The limitations of time and anisotropic spatial resolution, with lower spatial resolution in the direction of section selection, preclude the coverage of many intracranial arteries.
Hemodynamic analysis methods	Direct measurement of hemodynamic indicators	Combined with CFD	Combined with CFD	Combined with CFD	Combined with CFD
Common Parameter Comparison	MFV, PI, CVR, Velocity, CA	PR, Velocity, Velocity ratio, FPR, WSS, WSSR, SSR ratio, TDC	PR, Velocity, Velocity ratio, WSS, WSSR, CVR, SI, SIR, FSI	PR, WSS, WSSR, PB	PR, Velocity, Velocity ratio, Vorticity, WSS, WSSR, CFT, CCG

CFD, computational fluid dynamics; CTA, CT angiography; MRA, angiography; PPV, positive predictive value; NPV, negative predictive value; CTP, CT perfusion. DSA, digital subtraction angiography; CVR, cerebrovascular reactivity; CA, Cerebral Autoregulation; PR, pressure ratio; SSR, shear strain rates; TDC, Time-Density Curve; SI, signal intensity; FSI, Flow Signal Intensity; RFP, Relative Flow Presence; PB, Plaque Burden; SIR, signal intensity ratio; FPR, flow pattern ratio; WSS, wall shear stress; WSSR, wall shear stress ratio; SFS, Slow/Absent Flow Signal; CFT, Contrast Filling Time; CCG, Collateral Circulation Grading.

## 4 Discussion and future prospects

### 4.1 Advantages of hemodynamic analysis

#### 4.1.1 Non-invasive to blood vessels

Hemodynamic analysis techniques represented by CFD Modeling offers a relatively non-invasive way to minimize vascular disturbance for ICAS patients with severe stenosis of blood vessels. For the traditional gold standard DSA, measuring hemodynamic parameters in narrow areas through pressure wires increases direct stimulation at the stenotic site (16, 18, 96, 97). Invasive pressure guidewires may give rise to complications such as vascular perforation, vasospasm, and thromboembolism, with their operation being highly operator dependent. In contrast, non-invasive CFD reconstructions derived from data of CTA, MRA or DSA offer high reproducibility and patient safety, coupled with cost-effectiveness more suitable for routine clinical application, follow-up assessment, and large-scale screening. By reconstructing blood flow based on imaging data, CFD provides comparable diagnostic accuracy for important parameters without the direct introduction of instruments into stenotic arteries (91). TCD plays a significant role in the screening of ICAS patients by obtaining hemodynamic indices, and its operation is simple and minimally invasive. TCD also contributes to the acquisition of necessary boundary conditions in CFD modeling and simulation calculations (61). Although invasive methods remain indispensable in specific interventional scenarios, non-invasive hemodynamic evaluations are increasingly becoming a valuable supplementary tool, particularly in stroke risk stratification and treatment protocol formulation.

#### 4.1.2 Exploration of the pathogenesis of ICAS

Hemodynamic analysis has significantly deepened our understanding of ICAS pathogenesis. Hemodynamics has long been involved in the occurrence and development of atherosclerotic plaques in arteries. At the beginning of plaque development, low shear stress is believed to be the cause of atherosclerosis, while as the plaque progresses, high WSS is associated with the formation of vulnerable plaques (98–100). Serum metabolic markers such as creatinine, creatine, phenylalanine, mannose, paracetamol glucuronide, lactate, and apolipoprotein B (101) have also been demonstrated to play roles in the formation of ICAS. By integrating these metabolic factors with localized hemodynamic variables, investigators can explore how ICAS arises and progresses. This holistic view of ICAS pathobiology could guide future interventions aimed at stabilizing plaques or preventing their formation altogether.

#### 4.1.3 Etiological analysis of cerebral infarction

There are three main reasons for the occurrence of ischemic stroke caused by ICAS: hypoperfusion, artery-to-artery embolism, and plaque extension over small penetrating artery ostia (also known as branch atheromatous disease) (11, 102). Feng X’s research employed a CFD model based on CTA to investigate the relationships between the PR across the lesion, the WSSR, and stroke predominantly caused by artery-to-artery embolism (59). Li’s research investigates the relationship between PR and the types of infarctions at different junctional zones in intracranial atherosclerotic stenosis (103). Yin’s research integrates machine learning methodologies with hemodynamic analysis, effectively distinguishing the ischemic stroke mechanisms within the context of anterior circulation ICAS (104). Characterizing stroke etiology enables clinicians to tailor treatment, such as dual antiplatelet therapy or targeted hemodynamic support, thereby enhancing patient outcomes. Hemodynamic analysis is thus pivotal for distinguishing the specific cause of infarction and optimizing individualized management.

#### 4.1.4 Recurrence prediction and prognostic assessment of stroke

Hemodynamic analysis can be employed to investigate the factors associated with recurrent stroke and the prognostic risks in patients with ICAS. CFD modeling based on CTA, MRA, and DSA can be used to explore factors associated with stroke recurrence in patients with ICAS (56, 58, 60), The specific details have been previously elaborated upon in the preceding sections. Hemodynamic parameters obtained by TCD also have certain significance for the exploration of ICAS stroke recurrence (36). In the context of hemodynamic analysis concerning the long-term prognosis of ischemic stroke, Huang’s study demonstrates that both low PR and high WSSR are independently associated with recurrent ischemic stroke in the same territory (SIT) within 1 year. Notably, patients exhibiting both low PR and high WSSR exhibit a significantly elevated risk of SIT compared to those with normal PR and WSSR levels (57). Research on the long-term prognostic implications of hemodynamic parameters in ICAS is still relatively scarce. Future investigations might focus on novel hemodynamic metrics or enhanced analytical tools, including machine learning algorithms that integrate clinical and imaging data, to improve predictive accuracy and guide secondary prevention strategies.

#### 4.1.5 Endovascular therapy for ICAS

Hemodynamic analysis provides substantial benefits in planning and evaluating endovascular treatments. Zhou et al. employed CTA-based CFD to assess patients with severe posterior circulation stenosis before and after stenting, noting that successful recanalization significantly ameliorates hemodynamic parameters and regional perfusion (105). CFD modeling approaches can provide support and guidance for preoperative assessment of vascular structures and blood flow conditions, selection of stents (size and type), simulation of the stent implantation process, evaluation of post-implantation effects, and prediction of restenosis probability in patients with intracranial arterial stent implantation. By modeling anatomical and flow alterations post-implantation, CFD can help clinicians anticipate complications and refine postoperative management, underscoring its growing role in therapeutic decision-making for ICAS.

### 4.2 Challenges in ICAS hemodynamic analysis

#### 4.2.1 Willis circle and leptomeningeal collateral

Compared with extracranial arteries or cervical arteries, intracranial arteries have a more complex structure. The circle of Willis (CoW) plays a compensatory role in blood flow for patients with ICAS. When the structure of the CoW is incomplete (due to congenital deformities or acquired vascular stenosis and occlusion), the compensatory function of the CoW may be weakened, potentially promoting the occurrence of cerebral infarction in ICAS patients. Future research may conduct further subgroup analysis on ICAS cases under various completeness CoW conditions to eliminate the interference of compensatory blood supply. The leptomeningeal collaterals (LMCs) also plays a very important compensatory role in severe or even occlusive intracranial artery stenosis, but the hemodynamic evaluation of the meningeal collateral circulation is still in its infancy (61, 62). TCD is capable of measuring the blood flow velocities in the major cerebral arteries of specific patients, employing a simplified stepwise model to fully simulate the CoW. This model integrates such data to define boundary conditions (106). CTA utilizes time-density curves to assess LMCs (107). Strategically acquired gradient echo (STAGE) Magnetic Resonance Angiography employs a pair of refocused/defocused gradient echoes, which enhances image quality scores, improves signal-to-noise ratio, and reveals a greater number of LMCs (108). DSA serves as the gold standard for assessing collateral circulation, encompassing pathways from extracranial to intracranial regions, via the Willis circle, and through leptomeningeal vessels (109). The visualization of cerebral vasculature, particularly distal small vessels, exhibits variability contingent upon the volume and pressure of contrast medium injection (110). Although preliminary work has illuminated some aspects of LMCs hemodynamics, more advanced CFD studies are needed to quantify LMC capacity and elucidate its influence on stroke outcomes. The elucidation of LMCs pathways and the structural integrity of the CoW may contribute to the refinement of risk stratification for ICAS and inform personalized therapeutic strategies.

#### 4.2.2 Boundary conditions, transient and steady state

The ideal hemodynamic analysis for ICAS is characterized by individuality and instantaneity, and most of the parameters currently involved in the calculation of CFD models, such as boundary conditions such as distal flow resistance, inlet flow velocity, and pressure, are taken from the literature, which can lead to deviations between the simulation results and the actual patient-specific values. Relevant information can be found in Table 2. The setting of boundary conditions determines the reliability of CFD results; accurately obtaining these conditions is the goal pursued in CFD calculations. The current research consists of both transient (17, 85, 92, 96, 105, 111) and steady state (56–62, 71, 91, 103, 112–114) components. Although transient simulations better approximate physiological fluctuations, they are computationally demanding and typically require detailed *in vivo* measurements of flow velocity and pressure. Expanding sample sizes and harnessing advances in machine learning could bridge gaps in data acquisition and accelerate real-time hemodynamic modeling. Ultimately, refining boundary condition accuracy remains a critical step for maximizing the clinical relevance of CFD results.

**Table 2:** Research on the application of computational fluid dynamics modeling method imaging diagnoses for intracranial atherosclerotic stenosis.

Source image	Author	Year	Sample size	Anterior/posterior circulations	Inlet boundary condition	Outlet boundary condition	Steady/transient model	Relevant hemodynamic parameters	Clinical significance
CTA	Leng et al	2014	32	Anterior and posterior circulations	Set pressure = 120 mmHg	Set velocity = 60 cm/s.	Steady	PR, SSR ratio, velocity ratio	Exploring the relationship between hemodynamic analysis and the risk of stroke recurrence in ICAS.
CTA	Liu et al	2016	11	Anterior and posterior circulations	Pressure guidewire measurement	Calculated through the Windkessel model	Transient	FPR and FPRCFD	Investigating the efficacy of hemodynamic analysis in ICAS assessing severe intracranial stenosis.
CTA	Nam et al	2016	56	Anterior circulation	Define three systolic blood pressure (BP) ranges (109.2, 158, and 225 mmHg)	Set velocity = 60 cm/s.	Steady	PR, SSR ratio, velocity ratio	Exploring the relationship between vascular stenosis and hemodynamic changes in ICAS.
CTA	Leng et al	2018	85	Anterior circulation	Set pressure = 110 mmHg	According to TCD literature	Steady	Pressure gradient	Exploring the relationship between hemodynamics and LMC in ICAS.
CTA	Leng et al	2019	245	Anterior and posterior circulations	Set pressure = 110 mmHg	According to TCD literature	Steady	PR, WSSR	Exploring the hemodynamic patterns of sICAS stroke recurrence.
CTA	Feng et al	2020	157	Anterior and posterior circulations	Set pressure = 110 mmHg	According to TCD literature	Steady	PR	Exploring the hemodynamic patterns of sICAS stroke recurrence.
CTA	Lan et al	2020	83	Anterior circulation	Set pressure = 110 mmHg	According to TCD literature	Steady	PR	Exploring the relationship between hemodynamics and LMC in sICAS.
CTA	Lan et al	2020	39	Anterior and posterior circulations	Set pressure = 110 mmHg	According to TCD literature	Steady	rWSS measures	Investigating the hemodynamic mechanisms of sICAS progression/reversal.
CTA	Tian et al	2023	245	Anterior and posterior circulations	Set pressure = 110 mmHg	According to TCD literature	Steady	PR, WSSR	Exploring the hemodynamic patterns of sICAS stroke recurrence.
CTA	Raynald et al	2023	20	Anterior and posterior circulations	Pressure guidewire measurement	Iterative Calculation Approaching TCD Data	Transient	Pressure, velocity	Investigating the hemodynamic approach to assessing microvascular resistance in sICAS.
CTA	Feng et al	2023	99	Anterior circulation	Set pressure = 110 mmHg	According to TCD literature	Steady	PR, WSSR	Investigating the cerebral hemodynamic characteristics associated with AAE in symptomatic sICAS.
CTA	Li et al	2024	84	Anterior circulation	Set pressure = 110 mmHg	According to TCD literature	Steady	PR	Investigating the different mechanisms behind icas-induced infarction at the internal (IBZ) and cortical (CBZ) boundary zones.
MRA	Chen et al	2019	55	Anterior circulation	Blood flow calculated based on TCD measurements	Calculated through the Windkessel model	Transient	WSS, pressure drop	Exploring the relationship between ICAS vascular stenosis and hemodynamics.
MRA	Wu et al	2022	120	Anterior circulation	Calculating Blood Flow Velocity Based on Inlet Volume Flow Rate in a Healthy Patient	Mean arterial pressure at admission	Steady	PR, WSSR	Exploring the role of hemodynamic parameters in ICAS prediction models.
HR-MRI	Zhang et al	2021	40	Anterior circulation	Calculating Blood Flow Velocity Based on Inlet Volume Flow Rate in a Healthy Patient	Set the appropriate flow rate relationship based on the rate of blood flow distribution	Steady	PR, WSSR, WSS	Exploring the role of hemodynamics in the formation of intracranial atherosclerotic plaques and vascular remodeling.
DSA	Zhang et al	2022	51	Anterior circulation	Set velocity = 0.36 m/s	Set pressure = 0 Pa	Steady	c	Exploring the relationship between stents and hemodynamics in ICAS.
DSA	Zhou et al	2023	62	Posterior circulations	Set velocity = 50 cm/s	Set pressure = 0 Pa	Transient	Pressure, velocity, vorticity, WSS	Exploration of hemodynamic parameter changes in sICAS following stent treatment.
DSA	Wang et al	2023	18	Anterior and posterior circulations	Based on DSA simulation calculations.	Based on DSA simulation calculations.	Transient	PR	Exploring the relationship between hemodynamic parameters and perfusion in ICAS.
DSA	Yang et al	2024	121	Anterior and posterior circulations	Based on DSA simulation calculations.	Based on DSA simulation calculations.	Transient	PR	Exploring the evaluative significance of hemodynamic indicators in ICAS.

CFD, computational fluid dynamics; TCD, transcranial doppler; CTA, CT angiography; MRA, angiography; PPV, positive predictive value; NPV, negative predictive value; CTP, CT perfusion. DSA, digital subtraction angiography; PR, pressure ratio; WSS, wall shear stress; WSSR, wall shear stress ratio; SSR, shear strain rates; sICAS, symptomatic intracranial atherosclerotic stenosis; ICAS, intracranial atherosclerotic stenosis; ICAD, intracranial atherosclerotic disease, AAE, artery-to-artery embolism.

#### 4.2.3 Blood pressure fluctuations

Blood pressure is central to cerebral autoregulation and thus profoundly shapes hemodynamic states in ICAS. Liu J incorporates the blood flow and instantaneous blood pressure fluctuations of the Willis circle into a new model, and the hemodynamic parameters of the non-pressure guide wire calculated by this model are significantly correlated with those measured using a pressure guide wire (17). Feng X’s research found that low systolic blood pressure levels (≤130 mmHg) may be associated with a reduced risk of stroke recurrence in patients with normal perfusion pressure reactivity (PR), while in patients with inherently low PR, it may increase the risk of stroke (58). This could be related to the autoregulation of cerebral blood flow. When brain tissue is ischemic, it is necessary to appropriately increase systolic blood pressure levels to maintain normal cerebral perfusion. If it exceeds the regulatory range or the blood pressure is already low, it may increase the incidence of stroke. Nam discovered that lower blood pressure may lead to a pressure drop in distal measurements of cerebral flow divergence, while higher hematocrit levels may exacerbate the reduction in pressure among patients with severe ICAS. This research further confirms that variations in inlet pressure within boundary conditions can significantly impact the hemodynamics of ICAS (112). Consequently, ambulatory blood pressure monitoring, along with considerations of hematologic variables, should be integrated into future hemodynamic models to more accurately reflect the realities of patient care. However, the direct relationship between peripheral blood pressure (radial or brachial) and intracranial flow status remains incompletely understood and warrants further study.

## 5 Conclusion

In this review, we have presented the traditional methods used for diagnosing intracranial atherosclerotic stenosis (ICAS), analyzed the advantages and disadvantages of these methods, and illustrated examples of fluid dynamics analysis in various imaging techniques. Additionally, we have explored the prospects and concerns of applying fluid dynamics analysis techniques, such as computational fluid dynamics (CFD) modeling, in ICAS. As an emerging technology in recent years, fluid dynamics analysis is the collective achievement of developments in science, technology, and artificial intelligence fields. The application value of this approach in ICAS still requires exploration through a larger global sample and research centers. It is anticipated that its use will eventually be incorporated into the treatment guidelines for ICAS patients, offering new directions for the diagnosis and treatment of ICAS.

## Glossary

ICAS: intracranial atherosclerotic stenosis
TIA: transient ischemic attack
CFD: computational fluid dynamics
TCD: Transcranial Doppler ultrasound
CTA: computed tomography angiography
CTP: computed tomography perfusion
MRA: magnetic resonance angiography
HR-MRI: High-Resolution magnetic resonance imaging
VWMRI: Vessel wall magnetic resonance imaging
DSA: digital subtractive angiography
MCA: middle cerebral artery
BA: basilar artery
PR: the arterial pressure distal to the stenotic lesion to proximal pressure ratio
WSS: wall shear stress
WSSR: wall shear stress ratio
FFR: fractional flow reserve
CBF: cerebral blood flow
FPR: flow pattern ratio
LMC: leptomeningeal collateral
SIR: signal intensity ratio
